# An Entropic Mechanism of Generating Selective Ion Binding in Macromolecules

**DOI:** 10.1371/journal.pcbi.1002914

**Published:** 2013-02-28

**Authors:** Michael Thomas, Dylan Jayatilaka, Ben Corry

**Affiliations:** 1 Research School of Biology, Australian National University, Canberra, Australia; 2 School of Chemistry and Biochemistry, University of Western Australia, Perth, Australia; Max Planck Institute for Biophysical Chemistry Göttingen, Germany

## Abstract

Several mechanisms have been proposed to explain how ion channels and transporters distinguish between similar ions, a process crucial for maintaining proper cell function. Of these, three can be broadly classed as mechanisms involving specific positional constraints on the ion coordinating ligands which arise through: a “rigid cavity”, a ‘strained cavity’ and ‘reduced ligand fluctuations’. Each operates in subtly different ways yet can produce markedly different influences on ion selectivity. Here we expand upon preliminary investigations into the reduced ligand fluctuation mechanism of ion selectivity by simulating how a series of model systems respond to a decrease in ligand thermal fluctuations while simultaneously maintaining optimal ion-ligand binding distances. Simple abstract-ligand models, as well as simple models based upon the ion binding sites in two amino acid transporters, show that limiting ligand fluctuations can create ion selectivity between Li^+^, Na^+^ and K^+^ even when there is no strain associated with the molecular framework accommodating the different ions. Reducing the fluctuations in the position of the coordinating ligands contributes to selectivity toward the smaller of two ions as a consequence of entropic differences.

## Author Summary

Differentiating between Na^+^ and K^+^ ions is important for many cellular processes, such as nerve conduction and the regulation of membrane potentials. Different biological molecules utilise different methods to discriminate between ions. In this work, the reduced ligand fluctuation mechanism of ion selectivity is described. This entropy-driven mechanism is due to the limited thermal fluctuations of the atoms in some macromolecular ion binding sites. The elucidation of this mechanism offers a more complete picture of the ways in which the fundamental process of ion selectivity can be achieved.

## Introduction

The ability of some biological molecules to discriminate between different ions is crucial for their function. This differentiation is important, for example, in the generation (or regulation) of the action potential during cellular signalling, and the maintenance of an electrochemical gradient across the cell membrane [1]. Indeed, without this ability to discriminate between ions, a cell would quickly die. Of particular interest is how such molecules are able to distinguish between the monovalent cations Na^+^ and K^+^: these ions are both spherical, they have identical charges, and they differ in atomic radius by only 0.38 Å. It is incredible that some proteins, such as potassium selective ion channels, can discriminate between these two ions at nearly diffusion limited rates [2]–[5].

Although it is generally agreed that selectivity depends on a difference in free energy relative to bulk water of one ion compared to the other at some position within the transit pathway (i.e. how well the loss of free energy from dehydration is recouped by coordination with the protein), there are several different proposals which attempt to explain *how* this difference in free energy occurs. These proposals fall into three broad categories related to:

1. The ‘chemical nature’ of the ligands in the ion binding site or pore lining. By this it is meant the physical properties of the ligand, namely, its charge density distribution, polarisabilities, etc. These properties will dictate the balance of ion-ligand and ligand-ligand interactions [6]–[12]. A pore lined with carboxylate groups, for example, is likely to have different ion selectivity to a pore lined with carbonyl groups.
2. The number of ligands in the ion binding site. The number of ligands within a certain distance of the ion is assumed to be ‘enforced’ by the framework attached to the binding ligands (in biological systems, this will be the protein scaffold) [12]–[23]. Sites with particular ligand numbers can prefer to bind certain ions.
3. ‘Cavity effects’. By this it is meant some type of positional constraint on the atoms coordinating to the ion that yields a preference for binding one ion type over another. Although these positional restraints are also enforced by the scaffold or environment surrounding the ligands, they are more restrictive than enforcing just the number of coordinating ions as in that case the ligands may be free to move about the coordinated ion.

In this study we focus on the last category. To date, three different cavity effects have been proposed that can lead to ion selectivity: the ‘rigid cavity’, the ‘strained cavity’, and the ‘reduced ligand fluctuation’ (RLF) mechanisms. We discuss each in turn below; Table 1 summarises their key similarities and differences.

**Table 1:** A summary of the properties of the three cavity size mechanisms for creating ion selectivity.

Mechanism	Adjustable Ion-Ligand Distance	Penalty to Adjust Ion-Ligand Distance	Restricted Thermal Motion of Ligands
Rigid Cavity	no	N/A	yes
Strained Cavity	yes	yes	yes
Reduced Ligand Fluctuation	yes	no	yes

The ‘rigid cavity’ mechanism is perhaps the easiest to understand [24]–[26]. It suggests that ion selectivity is created by the ligand framework maintaining a certain *fixed position* (i.e. cavity size) about an ion regardless of the type of ion that is coordinated. Specific positions will be energetically more favourable for one ion type over another, thus contributing to selectivity for that ion. For example, when the smaller ion is favoured because the binding site is too small to fit the larger ion, this is often termed ‘size selectivity’. If the favoured ion is larger and sits more favourably in the cavity, this mechanism is commonly called ‘snug fit’. In reality the positions of the ligands will never be completely fixed, and their thermal motion is often larger than the size difference between Na^+^ and K^+^. Taking these thermal fluctuations into account, it has been demonstrated, in principle, that if the ligands fluctuate about some *fixed* average configuration for different ions this will create ion selectivity [10]–[12]. The question of which particular ion is selected by a given cavity site depends strongly upon the actual positions to which the ligands are constrained. Even this picture of a rigid cavity is probably too simplistic as the ligands are likely to fluctuate about different average positions when coordinating different sized ions. If the difference in the average positions is less than the difference in ion radii, one may still consider this situation to be a ‘rigid’ cavity. Our studies of many proteins suggest that the difference in average ion-ligand distance when coordinating Na^+^ and K^+^ is almost always similar to the difference in ionic radii, suggesting that a true rigid cavity is uncommon in proteins [12].

Unlike a ‘rigid cavity’, a ‘strained cavity’ allows for the average ion-ligand distances to adjust according to different ion types. However, in this case the adjustment comes at an energetic cost, called ‘strain’. Strain may be realised as a deformation within the ligand itself, or as a deformation of the ligand/protein scaffold, be it local [8], [27] or non-local to the ligand site [12], [19], [28]. Non-local strain may itself precipitate a conformation change in the protein (an extreme version of the effects of strain) thus further influencing ion selectivity [28]. A rigid cavity can be considered as an extreme form of a strained cavity, wherein the coordinating ligands resist any attempt to adjust to a new ion type in the binding site, perhaps due to an even larger cost in energy of deforming the protein scaffold. A continuum exists between the two, characterised by the degree of change in the average position of the ligands upon a change in ion type. As already noted, a rigid cavity is unlikely to exist in proteins, due to the inherent flexibility of these structures.

The idea that differentiation between ions could be achieved through a rigid cavity mechanism was first suggested by Mullins [24], [29], [30] who was investigating selectivity in ion channels. It was suggested that a rigid pore of an appropriate size could allow favourable interactions with K^+^, but be too big for Na^+^, leading to unfavourable interactions. This mechanism was supported voltage clamp experiments by Bezanilla and Armstrong [25] which suggested the pore was lined with backbone carbonyl oxygens, the particular arrangement of which mimicked bulk water more closely for K^+^ than Na^+^. More recently, Doyle *et al.* [26] have purported to suggest that ion selectivity in KcsA resulted from a rigid cavity mechanism. Whether it be a poor choice of words by the authors or misinterpretation by others (or a combination of the two), it seems that this explanation was offered as a caricature of a strained cavity mechanism, a point that is clarified in a later study [28]. The strained cavity mechanism has also been shown to play a role in ion selectivity in some ionophores, such as valinomycin [31]–[33], where the small amount of scaffolding between ion coordinating groups can leave the molecule sensitive to subtle sizes differences between coordinating ions. We propose that the rigid and strained cavity mechanisms are two domains of the same continuum; the term ‘strained cavity’ will herein be used to encompass this, except for when contrast between the two is required. Both share the common feature of requiring a resistance to changes in the positions of the coordinating ligands in order to generate ion selectivity.

Could ion selectivity be generated in an ion binding site through a ‘cavity’ like mechanism without the need for strain? In a previous investigation using simple abstract-ligand models it was noted that ‘cavity’ based mechanisms could still create selectivity even when both the cavity size is *not fixed* and when there is *no strain* associated with adjustment of the ligand positions [12]. In this case, the only ‘cavity’ factor controlling the binding energy of the ions was the degree of thermal motion associated with the ligands as the ligands were free to adopt their optimum positions for each ion type. This is the essence of the ‘reduced ligand fluctuation’ (RLF) model for ion selectivity that is the focus of this investigation.

Reduced ligand fluctuations (i.e. small values of root-mean-square (RMS) deviations in positions of the atoms forming the ligands from their average positions) of the ligands in the ion binding site compared to the rest of the protein were noted in atomistic molecular dynamics (MD) simulations of LeuT [12], [34], a leucine transporter, and Glt_Ph_, an aspartate transporter [12]. This situation contrasts with other molecules we studied previously, in which the RMS fluctuations of the ligands in the ion binding site were not notably smaller than the average across the protein [12]. It was also demonstrated, at least in the case of Glt_Ph_, that the Na^+^ binding sites were able to accommodate K^+^ (i.e. the sites were able to change the ion-ligand distance to a more favourable one for K^+^), ruling out a rigid cavity mechanism creating ion selectivity in this molecule. It could be the case that there is an energetic penalty in adjusting to K^+^ (strain), however, this is difficult to quantify [12], [18].

In this investigation we try to better understand how reducing ligand fluctuations creates ion selectivity, improving on the simple models used in previous work [12] by using better force field parameterisations, and by investigating a greater variety of model systems. Simplified model systems have been used to study ion selectivity in a range of molecules including potassium channels [8], [9], [12], [13], [18], [19], [22], [27], a sodium channel [35], NaK channels [27] and kainate receptors [36]. In addition, the previous work never addressed exactly *how* reducing the thermal motion of the ligands leads to selectivity. Using the more detailed model systems, and by analyzing binding-energy components, we are able to propose an answer here. Of course, the RLF mechanism is not mutually exclusive with the other means of obtaining ion selectivity previously mentioned; it may work in concert with these other effects. However, in this investigation, we wish to study it in isolation, so as to clearly discern it from these other possibilities.

## Methods

### Theory

One can elucidate the effect of the RLF mechanism on the selectivity of an ion binding site by investigating how a series of ‘abstract’ model systems (pioneered by Noskov *et al.*) [8] respond to a reduction in the RMS fluctuation on the coordinating ligands. These model systems consist of a number, 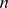, of abstract ligands (in this case based on formaldehyde), where each oxygen atom is confined to a 3.5 Å sphere about an ion, either Li^+^, Na^+^ or K^+^. This first constraint is enforced by a one-sided harmonic potential with a very large force constant. This spherical constant is not meant to precisely define the coordination numbers of the ions, but rather to control the number of ligands near to the ion as a model of the composition of a biological ion binding site. The position of the central ion is fixed, while each atom in the coordinating ligands can be further constrained by placing them in an additional harmonic potential, 
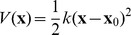
 centered at a nominated position. The amount of thermal fluctuation of the ligands can be controlled by altering the force constant, 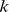. The choice of physical location at which the harmonic restraint is placed is very important. In order to isolate RLF from strain, the harmonic restraint needs to be placed at an ‘optimal’ ion-ligand distance for each ion type. This position is defined as the maximum of the first peak in the radial distribution function from dynamics simulations of Li^+^, Na^+^ or K^+^ surrounded by 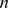 ligands with no harmonic restraint. The systems where the position of the coordinating ligands are controlled are referred to as 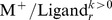 with 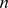 ligands, where M^+^ = Li^+^, Na^+^ or K^+^, with geometries determined by the vertices of optimal coordination polyhedra for n = 4,6,8 and by packing circles on spheres for n = 5,7 [37]. 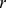 is the distance between the center of the ion and the center of the coordinating atom.

In order to quantify ion selectivity, the free energy to exchange two ions between bulk water and our model binding sites is calculated. As each model system is allowed to adopt an ‘optimal’ cavity size (or ion-ligand distance) for each ion type, a series of free energy perturbation molecular dynamics (FEP MD) simulations are required in order to describe the contribution to ion selectivity from this mechanism in a meaningful way.

The overall reaction to be investigated is an exchange reaction of the ions 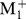 and 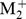 between an optimally sized hypothetical model system for each ion (at ion-ligand distances 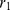 and 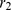) with controllable fluctuations and bulk water:
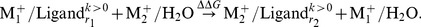
(1)To calculate 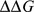 and other binding energies we begin by effectively ‘morphing out’ the positional constraint on the ligands around one of the ions, 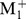. This is achieved by having two sets of ligands in the simulation: one set where each atom is subjected to a harmonic constraint (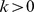) and one with no harmonic constraint (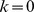).
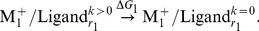
(2)The system with no harmonic constraint, 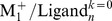 (but still with a 3.5 Å radial constraint) can now undergo an exchange reaction with another ion 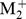 bound in bulk water:
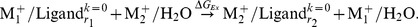
(3)This exchange reaction consists itself of two separate FEP MD simulations:
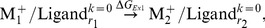
(4)

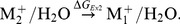
(5)The values for 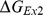 used here were calculated from the free energies of solvation by Joung and Cheatham [38]. Now the constraints can be morphed into the model system containing 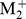:
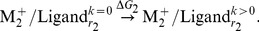
(6)The change in exchange free energy for the overall reaction is then given by
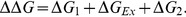
(7)

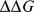
 will be positive if 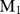 is thermodynamically preferred in the ion binding site and negative if 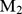 is preferred. This quantity can also be studied as the thermal fluctuations (i.e. the value of 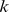) is reduced without reference to the energy involved in bringing the ion into the site from the bulk. The contribution to ion selectivity due only to the RLF mechanism alone, 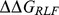, can be defined as:
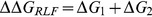
(8)


### Abstract Models

Abstract model systems consisted of 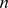 abstract ligands (based on formaldehyde with partial charges of carbon +0.5, oxygen −0.5 and hydrogen 0.0) where each oxygen atom is confined to a 3.5 Å sphere by use of a spherical flat-bottomed, steep harmonic potential constraint. Reducing the ligand fluctuations was achieved by confining each atom to a harmonic potential, varying the force constant, 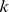, from 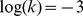 to 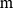 in 0.5 increments. This harmonic potential was placed at the vertices of optimal coordination polyhedra for 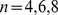, and at geometries governed by packing circles on spheres for 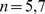 [37], at a distance from the ion corresponding to the first peak in the radial distribution function of a harmonically unconstrained (but still with the 3.5 Å spherical constraint) simulation determined for each ion type. A harmonic restraint is used for this purpose as a first order approximation; the restraining potentials exhibited in nature would probably be somewhat anharmonic and anisotropic. Two sets of ligands, i.e. 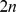, are required in order to conduct FEP MD between harmonically constrained and harmonically unconstrained ligands with one set annihilated and the other set exnihilated during the simulation. However, both endpoints represent 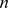 ligands coordinating to an ion. Errors in 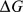 were minimised by using a large number of 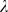 windows, with 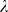 values of 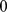, then 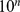 for 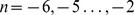 then 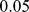 then incrementing by 0.05 to 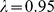, then 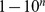 for 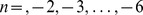 then 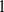. Forward and reverse morphs were conducted for each ion/model/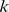 combination. The maximum difference in the forward and reverse morphs for 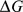 was 0.94 kcal/mol, with an average difference of 0.27 kcal/mol. The maximum error in 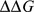 (the summation of errors from 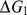, 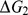 and 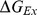) is estimated to be 1.1 kcal/mol with an average error of 0.62 kcal/mol. Energies were averaged over 4 ns for each 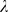 window. Softcore potentials were utilised using a van der Waals radius shift coefficient of 1. A cut-off distance of 12 Å and a switching distance of 10 Å is used for electrostatic and van der Waals interactions.

FEP MD simulations where the ion is being morphed used only one set of ligands, as the ligands are not bound by a harmonic constraint. 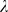 was varied from 0 to 1 in 0.05 increments. Energies were averaged over 4 ns for each 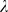 window. Softcore potentials were utilised using a van der Waals radius shift coefficient of 1. A cut-off distance of 12 Å and a switching distance of 10 Å is used for electrostatic and van der Waals interactions. All simulations were conducted using NAMD2 [39] with the CHARMM27 force field [40] at 310 K with 1 fs timesteps. Force field parameters for Li^+^, Na^+^ and K^+^ are from Joung and Cheatham [38].

The volumes occupied by the coordinating ligands were calculated using the VolMap tool in VMD [41], with a resolution of 0.1 Å, and an in-house Fortran program with an isosurface value of 1.0.

### Amino Acid Transporter Models

FEP MD simulations were conducted identically to the harmonically constrained abstract models and the harmonically unconstrained models discussed in the previous section. To set up these systems the positions of the atoms coordinating to Na^+^ in each ion binding site, along with the atoms directly bonded to these and the ions themselves were extracted from the crystal structures of Glt_Ph_, PDB accession code 2NWX [42], and LeuT, PDB accession code 2A65 [43]. These four model sites were then energy minimised with Li^+^, Na^+^ and K^+^ present as the central ion. These minimised structures provided the initial starting coordinates for further simulations along with the coordinates to which the harmonic constraints were placed (i.e. 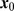).

### 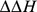 and 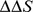



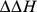
 was calculated by extracting the average total potential energy from the first and last window of each FEP MD simulation and combining them in a fashion as described for 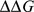 in the theory section. 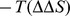 was calculated for each 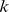 using 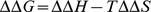.

## Results/Discussion

### Abstract Models

**Figure 1:**
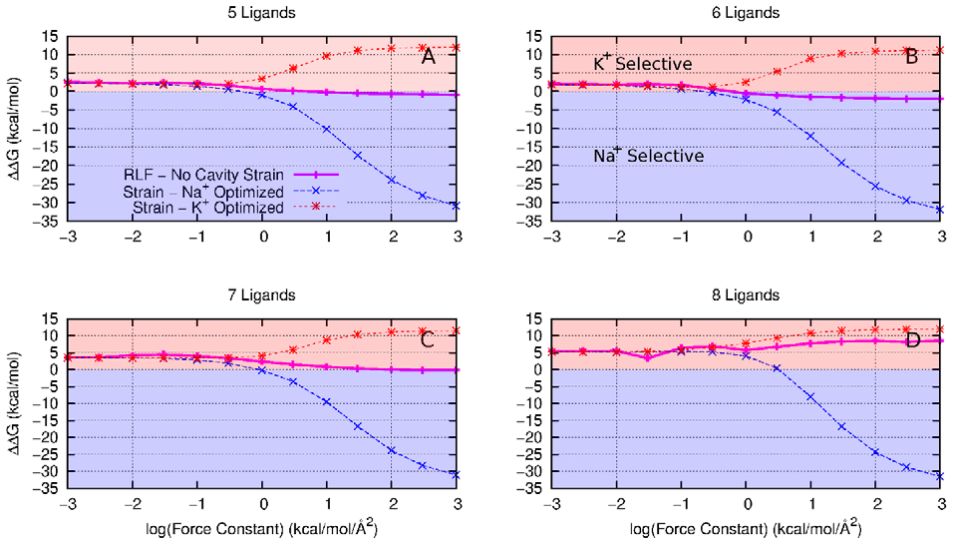
The effect of reducing the size of the ligand thermal fluctuations on selectivity between Na^+^ and K^+^ (solid magenta line), controlled by increasing harmonic constraint constant 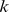
 **, for (A) 5-fold, (B) 6-fold, (C) 7-fold and (D) 8-fold coordination states.** These are compared with a strained cavity model at ion-ligand distance optimised for Na^+^ (dotted blue line) and K^+^ (dotted red line). A negative value (blue region) of 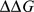 indicates the model site is selective for Na^+^, a positive value (red region) indicates K^+^ selectivity. The non-zero value of selectivity when 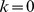 is due to the chemical nature and number of ligands as discussed elsewhere [12].

**Figure 2:**
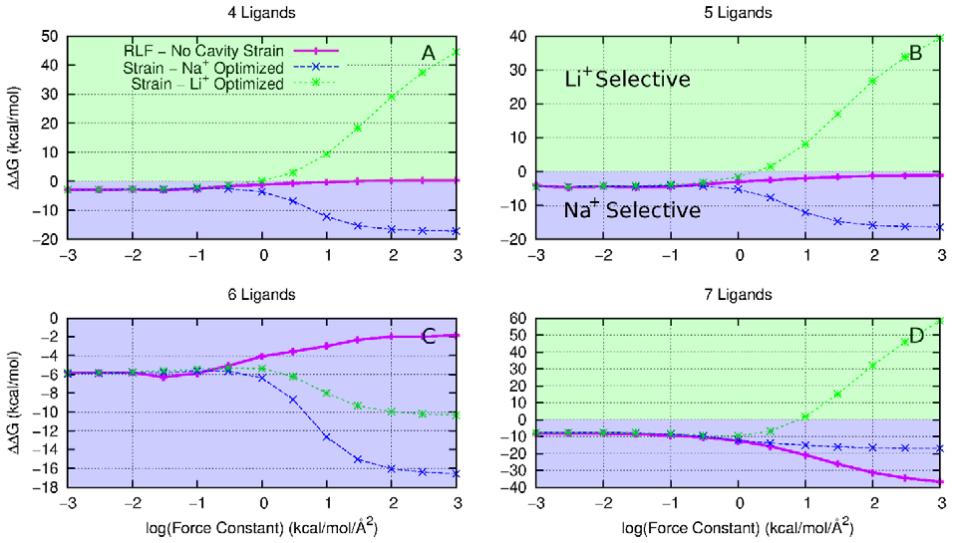
The effect of reducing the size of the ligand thermal fluctuations on selectivity between Li^+^ and Na (solid magenta line) with increasing harmonic constraint constant, 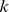
 **for (A) 4 fold, (B) 5 fold, (C) 6 fold and (D) 7 fold coordination states.** These are compared with a strained cavity model at ion-ligand distance optimised for Li^+^ (dotted green line) and Na^+^ (dotted blue line). A negative value (blue region) of 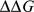 indicates the model site is selective for Na^+^, a positive value (green region) indicates Li^+^ selectivity.

The ion selectivity of the abstract models, including contributions from the RLF mechanism, is plotted versus the force constant, 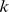, for binding sites with 4–8 ligands in Fig. 1 and 2. For comparison we also plot two sets of results for the strained cavity mechanism, that is, when the same location of the restraint is used for both ions rather than using an ‘optimal’ position for each ion type.

Fig. 1 demonstrates that each abstract model system displays an inherent selectivity for K^+^ over Na^+^ when there is little or no restraint on the fluctuation of the ligands, in line with results from previous studies by ourselves [12] and others [8]. It must be stressed that this inherent selectivity is only for this particular type of ligand. Naturally, as 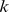 increases the strained cavity models become more selective for the ion to which the positions of the ligands are optimised (blue, Na^+^, and red, K^+^, lines in Fig. 1). This effect can be quite large (tens of kcal/mol) and tends to plateau for the largest values of 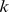 tested in this study, where the strained cavity becomes a ‘rigid’ cavity.

The selectivity of the abstract models for Na^+^/Li^+^ is a little more complicated than for Na^+^/K^+^. Each abstract model displays an inherent selectivity for Na^+^ when there is little or no positional constraint on the coordinating ligands, as Fig. 2 shows. The effects of introducing the strained cavity begin to show as the strength of the restraint increases; the positions to which the ligands are constrained determine the selectivity (green, Li^+^ and blue, Na^+^, lines). However, there is some anomalous behavior, especially for the six ligand case (Fig. 2 C) where strong restraints at both Li^+^ and Na^+^ optimised positions increase selectivity toward Na^+^.

**Figure 3:**
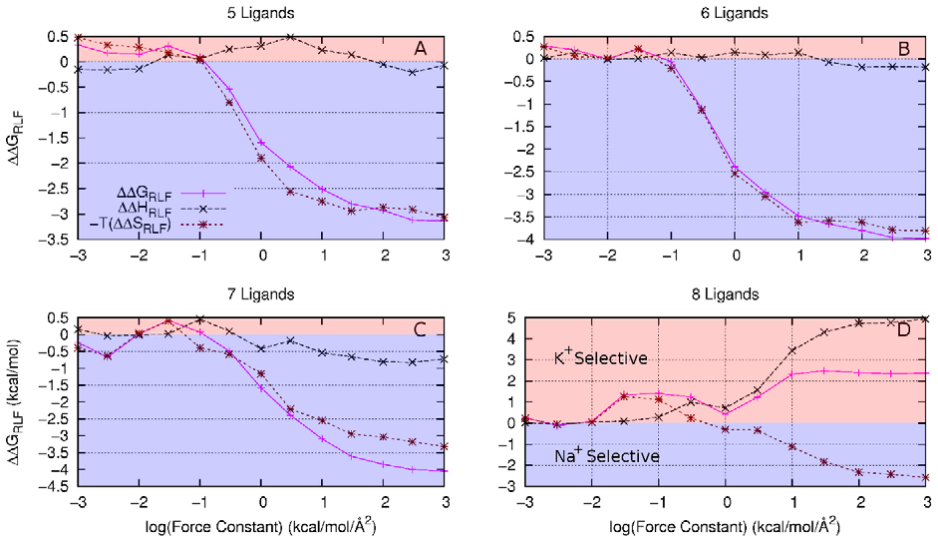
A decomposition of 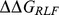
 **(magenta), in the absence of cavity strain, into** 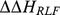 **(black) and** 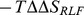 **(brown) components of ion selectivity between Na^+^ and K^+^ for (A) 5 fold, (B) 6 fold, (C) 7 fold and (D) 8 fold coordination states.** The red region indicates a contribution toward K^+^ selectivity, while the blue region indicates a contribution toward Na^+^ selectivity.

A more subtle situation arises when the position of the restraint is different for each ion, i.e. when we consider the RLF mechanism without any strain. Although the difference looks small on the scale of Fig. 1, a 2–5 kcal/mol increase in selectivity toward Na^+^ occurs for the exchange reaction with K^+^ with 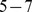 ligands when the size of the thermal fluctuations is reduced (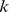 increased). The majority of this change occurs for 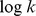 between 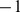 and 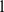, plateauing for 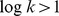 (see Fig. 3 to see this plotted in a different scale). This change in ion selectivity alters these models from K^+^ selective sites to Na^+^ or non-selective sites. For 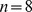, the selectivity in the already K^+^ selective site is further enhanced by 2–3 kcal/mol.

A similar situation arises in the exchange reaction between Li^+^ and Na^+^ (Fig. 2). Again, the most drastic changes occur for the strained cavity model as 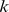 increases. The changes in selectivity due the RLF mechanism are again smaller than those for the strained cavity but the trends are similar to that seen for Na^+^/K^+^ in the cases with 4 or 5 ligands, with increasing 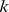 moving selectivity toward Li^+^. The situation with 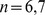 is more confusing with a reduction in fluctuations causing selectivity toward Na^+^ for 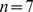. The 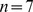 model also has a much larger change in 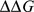 (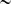
29 kcal/mol) than the 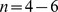 models (3–4 kcal/mol). Also, the RLF results in the 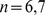 models do not fall within the bounds of the strained cavity results.

### How Does the Reduced Ligand Fluctuation Mechanism Create Ion Selectivity?

**Figure 4:**
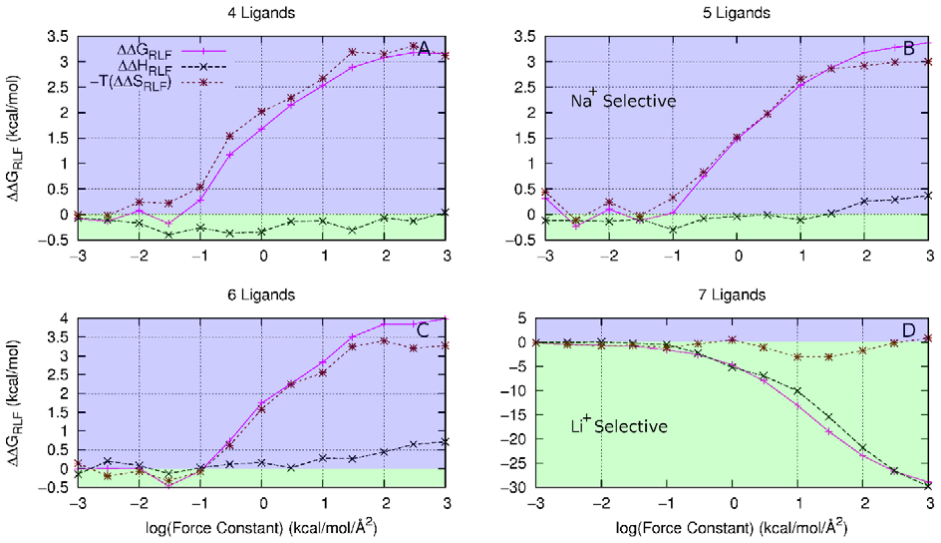
A decomposition of 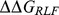
 **(magenta), in the absence of cavity strain, into** 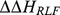 **(black) and** 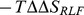 **(brown) components of ion selectivity between Na^+^ and Li^+^ for (A) 4 fold, (B) 5 fold, (C) 6 fold and (D) 7 fold coordination states.** The green region indicates a contribution toward Li^+^ selectivity, while the blue region indicates a contribution toward Na^+^ selectivity.

**Figure 5:**
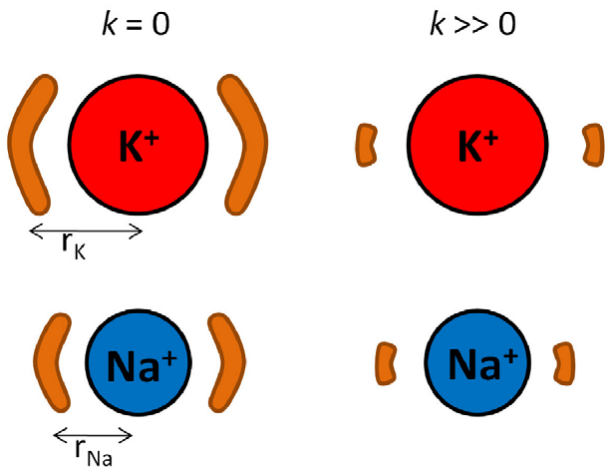
A depiction of the origin of selectivity via the RLF mechanism. Selectivity arises from the difference in the entropy change from unconstrained ligands (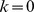) to constrained ligands (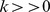) between K^+^ and Na^+^. The brown regions represent the volume in which two ligands can move about the ion. As the ligands' fluctuations become constrained, the ligands experience a greater decrease in available volume and thus entropy when coordinating a large ion than when coordinating a smaller ion.

Having shown that restricting the fluctuations in the positions of the ligands creates selectivity for one ion over another even in the absence of strain, the question remains, how does this occur? If we decompose 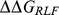 into the enthalpic, 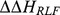, and entropic, 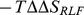, components the driving force behind this change in selectivity becomes apparent. In the exchange between Na^+^ and K^+^ (Fig. 3) for 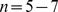 and Na^+^ and Li^+^ (Fig. 4) for 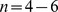, the 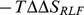 contribution follows very closely with 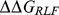 indicating this selectivity is largely due to entropy differences. Intuitively one would expect the change in the available number of states (as you decrease the allowed fluctuations) to be largest for the larger ions for the following reasons. The number of possible configurations for coordination in the 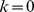 (only bound by a 3.5 Å constraining sphere) is greater for the larger ion than the smaller ion because of the greater volume available at the larger ion-ligand distance, as depicted in Fig. 5. As the positional restraint is increased (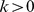), the number of states become approximately equal for different ion types. Hence the change in entropy between a non-restrained and restrained system is largest for the larger ion. This can be shown to be the case by considering the difference in volume sampled by the coordinating oxygen atoms as their fluctuations becomes more and more constrained. For instance, this change in volume for the four fold coordination state is 3640 Å^3^ for Li^+^, 5050 Å^3^ for Na^+^ and 5820 Å^3^ for K^+^ when comparing 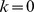 and 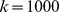. Reducing the thermal fluctuations on the ligands causes a greater decrease in entropy when they coordinate a larger ion compared to a small one. As a consequence, this reduction of thermal fluctuations favours small ions binding in the site.

A different situation arises with 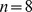 for K^+^/Na^+^ systems and 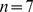 for the Li^+^/Na^+^ systems. In the former both the enthalpic and entropic terms play a role, while the latter is dominated by the enthalpic contribution. Analysis of these situations shows that the reason for the different behaviour is due to the difficulty in packing a large number of ligands around the smaller ions. As the 3.5 Å constraining sphere does not precisely define the coordination numbers, it is possible for ligands to form a second coordination shell about the small ions when the number of ligands is large. Increasing the force constant, 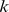, brings all the ligands to a uniform distance, yielding enthalpic changes in addition to the entropic changes seen for the cases with fewer ligands.

### Glt_Ph_, LeuT and KcsA Model Systems

The motivation for proceeding with this investigation was the realisation that in at least two amino acid transporters, the aspartate transporter, Glt_Ph_, and the leucine transporter, LeuT, the ligands forming the two Na^+^ binding sites display reduced RMS fluctuation in their positions compared to similar atom types elsewhere in the protein. The RMS fluctuation of the oxygen atoms forming the four sites ranged between 0.3 and 0.5 Å, whereas the other oxygens in the protein had values larger than 0.7 Å [12], [34]. This is thought to be the result of extensive hydrogen bonding networks in the vicinity of the ion binding sites, as shown to be the case with one of the LeuT sites [44]. Additional constraint may be imparted upon the coordinating ligands if they belong to an amino acid in a more rigid secondary structure, such as backbone carbonyl oxygens of 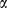-helices. The ion binding sites in both LeuT and Glt_Ph_ contain many of these backbone carbonyl oxygen atoms (table 2). More generally, there may also be sterical effects that limit the motion of the coordinating ligands.

**Table 2:** Composition of the amino acid transporter model ion binding sites.

LeuT		Glt_Ph_	
Na1	Na2	Na1	Na2
LEU:carboxylate	GLY20:carbonyl	GLY306:carbonyl	THR308:carbonyl
ALA22:carbonyl	VAL23:carbonyl	ASN310:carbonyl	ILE309:carbonyl
ASN27:carbonyl	ALA351:carbonyl	ASN401:carbonyl	SER349:carbonyl
THR254:carbonyl	THR354:hydroxyl	ASP405:carboxylate*	ILE350:carbonyl
THR254:hydroxyl	SER355:hydroxyl		THR352:carbonyl
ASN:carbonyl			

* indicates that both oxygen atoms are present in the carboxylate group.

Attempts have been made to explain the Na^+^ selectivity in LeuT. Yamashita *et al.* [43] suggested that it could be a result of a more snugly fitting site for Na^+^ than the larger K^+^, which would upset hydrogen bonding or packing interactions in the protein. This is in line with the strained cavity mechanism described by Lockless *et al.* [28] in K^+^ channels. Other investigations suggest that the first binding site (Na1) achieves Na^+^ selectivity over both Li^+^ and K+ due to the strong electrostatic interactions resulting from the coordinating carboxylate ligands, while the second binding site (Na2) achieves this through a strained cavity mechanism [10], [44]. To the best of our knowledge, similar investigations have not been undertaken for Glt_Ph_. Therefore, we investigate the effect on ion selectivity of reducing the fluctuations in the ligands forming each binding site in the transporters by constructing corresponding model systems.

The model sites for Glt_Ph_ were constructed from the outward facing, Na^+^ and aspartate bound crystal structure [42]. Only two (Na1 and Na2) of the three Na^+^ binding sites are considered, as the exact nature of the third is still under debate [45]–[47]. Models for the two LeuT Na^+^ binding sites were constructed from the Na^+^ and leucine bound crystal structure [43]. For each model, the coordinating atom, and atoms bonding directly to these, were used to construct simple dipolar ligands in order to model the electrostatic environment experienced by the bound ion. The composition of each model is detailed in table 2. The initial coordinates of these atoms were taken from the crystal structure and then allowed to energy minimise with Li^+^, Na^+^ and K^+^ independently. This gave us the final optimal coordinates for each ion type at which harmonic constraints were applied. Simulations were conducted to investigate RLF as described earlier for the abstract ligand models.

**Figure 6:**
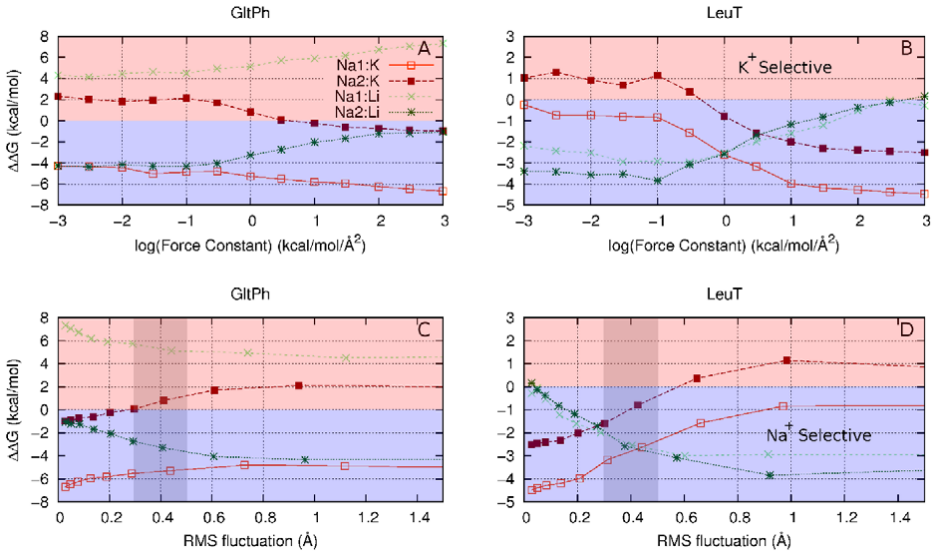
The effect of reducing the fluctuations of the ligands in the amino acid transporter model sites. (A) Na^+^ in the Glt_Ph_ model sites Na1 and Na2 being morphed to K^+^ (orange and dark red) or Li^+^ (light green and dark green). (B) Na^+^ in the LeuT model sites Na1 and Na2 being morphed to K^+^ (solid orange and dotted dark red) and Li^+^ (dotted light green and solid dark green). (C) The same free energies as (A) plotted against RMS fluctuations. The grey area corresponds to the observed RMS fluctuations in full system simulations of Glt_Ph_ conducted in other studies [12], [34]. (D) The same free energies as (B) plotted against RMS fluctuations. Negative values (blue region) indicate Na^+^ selectivity, positive values (red regions) indicates K^+^ or Li^+^ selectivity.

**Figure 7:**
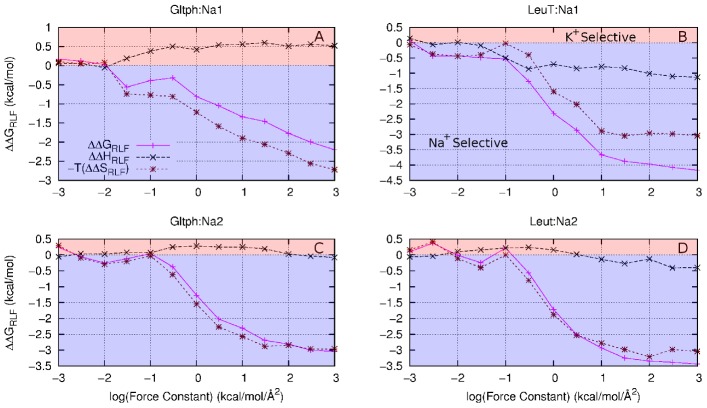
Decomposition of 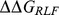
 **(magenta) into** 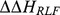 **(black) and** 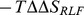 **(brown) for the Na^+^** 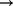 **K^+^ morph for (A) Glt_Ph_:Na1, (B) Glt_Ph_:Na2, (C) LeuT:Na1 and (D) LeuT:Na2.** Negative values (blue region) indicate Na^+^ selectivity, positive values (red region) indicates K^+^. Similar plots for the Na^+^ 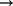 Li^+^ situation are given in Text S1.

As the amount of allowed fluctuation in the ligands of the amino acid transporter models are reduced (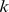 increased), the change in the free energy of the exchange reaction between two ions (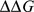) behaves in a very similar manner to the abstract models; the decrease in fluctuation contributes selectivity to the smaller of the two competing ions (Fig. 6). If we recall that the most of the oxygen atoms in Glt_Ph_ and LeuT displayed RMS fluctuations greater than 0.7 Å, we see from Fig. 6 C and D that there is little to no contribution toward ion selectivity in this region. However, this contribution becomes significant for RMS fluctuation values observed for the oxygen atoms at the ion binding sites (the grey regions in Fig. 6 C and D). In this model, the ligands are able to adopt a preferred ion-ligand distance, and at no energy cost (in contrast to the strained cavity mechanism), yet a degree of ion selectivity is still created by the reduction in ligand fluctuation. A decomposition of the free energy change in each of the sites into the enthalpic and entropic contributions clearly demonstrate that this effect is primarily a consequence of entropy differences (Fig. 7).

It is evident from the non-zero values of 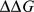 at large RMS fluctuations (small 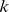) of the ligands in Fig. 6 that the chemical nature of the ligands and/or coordination numbers play a role in creating ion selectivity in the ion binding sites of LeuT and Glt_Ph_. As the RMS fluctuations decrease, the contribution from RLF merely adds to this. Nevertheless, in the absence of a strained cavity, it is crucial for enhancing selectivity for Na^+^ over K^+^ in LeuT. While a strained cavity and the chemical nature of the ligands may play a role in creating selectivity in themselves, we hope to show here that the observed selectivity could also involve a contribution from the RLF mechanism.

Note that the 8 ligand coordinated Na^+^/K^+^ abstract model is very similar to the crude model S_2_ K^+^-selective binding site in the selectivity filter of the potassium ion channel KcsA investigated previously by Thomas *et al*. [13] There is a very slight to no increase (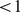 kcal/mol towards K^+^ selectivity) in the region corresponding the RMS fluctuations (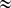
0.75 Å) [8] of the filter, suggesting that the RLF mechanism does not play a role in KcsA. Of course this result for KcsA depends on Na^+^ and K^+^ binding at the same sites in the selectivity filter; a view which has been challenged with the proposal of distinct binding sites for the two ions [48]–[50].

It should be noted that our study of the RLF mechanism and previous work by Yu *et al.* [10] differ in one very significant way. In the latter, constraints are placed at the crystallographic coordinates for the Na^+^ model binding sites for LeuT and the K^+^ model binding site for KcsA. This means that there is only one set of constraint positions for both ion types (Na^+^ and K^+^) and thus their analysis includes the influence of a strained cavity, which is to say that a change in enthalpy, as well as entropy, will influence selectivity. While we do not deny that such an effect may play a role, we have isolated the RLF mechanism by allowing the ligands to freely adapt to each ion type. This means that the positions of the constraints on the ligands are optimal for each ion type, eliminating any ‘strained cavity’ effect from this analysis.

The simple models of the ion binding sites in LeuT and Glt_Ph_ were not designed so as to quantitatively reproduce experimental and more detailed simulation results, only to show how the RLF mechanism may influence the overall selectivity. Other factors may become important when considering the selectivity of the protein as a whole, such as the coupling between the ion selective sites [51]. However, these simple models are able to qualitatively reproduce experimental [43] and more detailed simulation [44] results for Na^+^/K^+^ selectivity in LeuT. In fact, Na2 changes from a K^+^ to a Na^+^ selective site when the reduction in the ligand fluctuations are accounted for. When compared to experimental [42] and more detailed simulations [12], Na^+^/K^+^ selectivity in Glt_Ph_ is qualitatively reproduced for Na1, while Na2 is rendered essentially non-selective with the RLF effect. A table comparing results from this study to experimental and more detailed simulations can be found in text S1.

What conclusions can we draw from this? Given that the amino acid transporter model binding sites are exceedingly simple, any conclusion drawn will be tentative. However, even though these models may be crude, they do demonstrate that reducing the fluctuation of the coordinating ligands, can affect ion selectivity even if there is no strain in the protein. Again it is shown that the RLF mechanism is primarily a consequence of entropy differences. As this mechanism relies heavily on entropic factors, experimental investigations into the temperature dependence of ion selectivity in these amino acid transporters could perhaps shed further light on its role in biological systems.

### Conclusion

Reducing the thermal fluctuation in the positions of the coordinating ligands affects the binding of Li^+^, Na^+^ and K^+^ differently and is able to contribute toward ion selectivity, even when there is no strain associated with the protein adapting to different ions. This contribution to ion selectivity is due to entropic differences arising with different ions in the site, resulting from the larger difference in accessible states for the ligands surrounding the larger ions than the small ones when the thermal fluctuations are reduced. Thus, this mechanism of ion selectivity favours of small ions over larger ions.

## Supporting Information

Text S1 Supporting text (Text S1) is available giving further information on the nature of the model binding sites, and additional results showing the free energy values for both forward and reverse FEP morphs.
(PDF)
