# Supplementary material for: An Entropic Mechanism of Generating Selective Ion Binding in Macromolecules
Source: PLoS Comput Biol. 2013 Feb 28;9(2):e1002914. doi: 10.1371/journal.pcbi.1002914 (PMC3585399; doi:10.1371/journal.pcbi.1002914)
Supplement: Text S1 — Supporting text (Text S1) is available giving further information on the nature of the model binding sites, and additional results showing the free energy values for both forward and reverse FEP morphs. (PDF) [file pcbi.1002914.s001.pdf]

Text S1

Michael Thomas[1,*], Dylan Jayatilaka[2], and Ben Corry[1]

[1]Research School of Biology, Australian National University, Canberra, Australia

[2]School of Chemistry and Biochemistry, University of Western Australia, Perth, Australia

[*]Corresponding Author, E-mail: michael.thomas@anu.edu.au

Model Type	Binding Site	Ions Studied	Number of Atoms
Abstract Model	4 Ligands	$Li^+/Na^+/K^+$	33
Abstract Model	5 Ligands	$Li^+/Na^+/K^+$	41
Abstract Model	6 Ligands	$Li^+/Na^+/K^+$	49
Abstract Model	7 Ligands	$Li^+/Na^+/K^+$	57
Abstract Model	8 Ligands	$Li^+/Na^+/K^+$	65
LeuT	Na1	$Li^+/Na^+/K^+$	27
LeuT	Na2	$Li^+/Na^+/K^+$	25
Glt_{Ph}	Na1	$Li^+/Na^+/K^+$	19
Glt_{Ph}	Na2	$Li^+/Na^+/K^+$	21

Table S1: A description of the composition of each system investigated in this study. The number of atoms includes annihilated and exnihilated atoms.

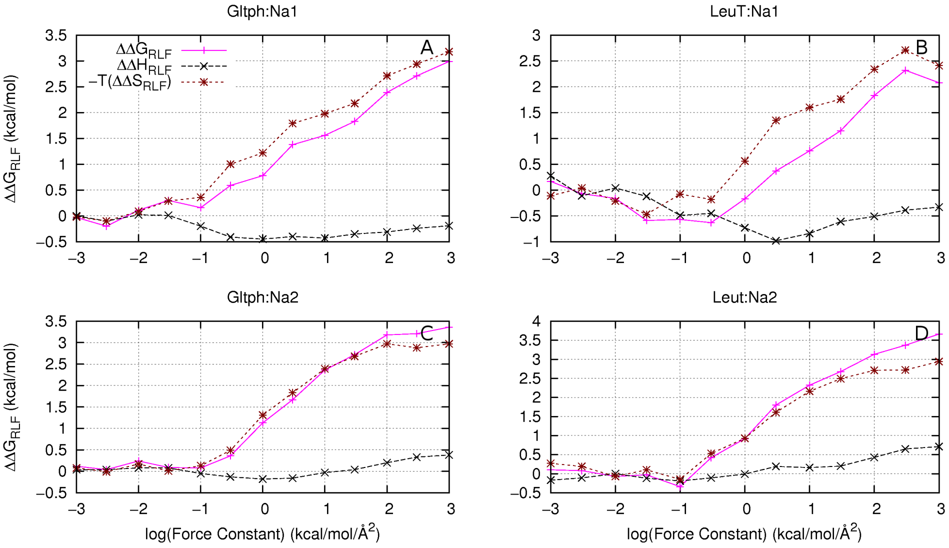

Figure S1: Decomposition of $\Delta\Delta G_{RLF}$ (magenta) into $\Delta\Delta H_{RLF}$ (black) and $-T\Delta\Delta S_{RLF}$ (brown) for the $Na^{+} \rightarrow Li^{+}$ morph for (A) $\mathrm{Glt_{Ph}}$:Na1, (B) $\mathrm{Glt_{Ph}}$:Na2, (C) LeuT:Na1 and (D) LeuT:Na2. Negative values indicate Na^{+} selectivity, positive values indicates K^{+}.

Binding Site	This Work	Experimental	Detailed Simulation
LeuT:Na1	-2.7	<-5 [1]	-6.1 [2]
LeuT:Na2	-1.0	<-5 [1]	-3.2 [2]
$\mathrm{Glt_{Ph}}$	-5.2	<-3 [3]	-1.3 [4]
$\mathrm{Glt_{Ph}}$	-0.9	<-3 [3]	-0.6 [4]

Table S2: A comparison of the ion selectivity free energies from this study, experiment and full system simulations (includes entire protein imbedded in a lipid bilayer, immersed in a salt solution). All energies are in kcal/mol.

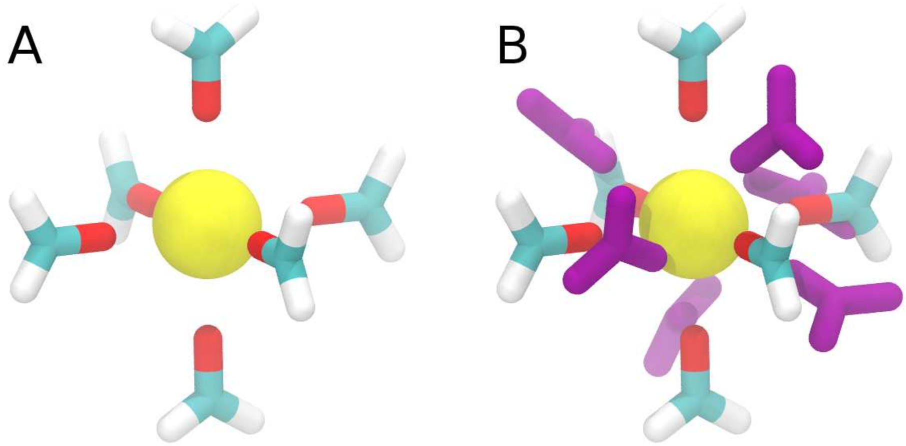

Figure S2: An example of the abstract models used to investigate the RLF mechanism. Na^+ is represented in yellow, carbon in cyan, oxygen in red and hydrogen in white. (A) A snapshot of the 6 ligand system with $k = 1000$. (B) A snapshot of the same system both sets ($k = 1000$ and $k = 0$ (magenta)) of abstract ligands shown. One set is annihilated while the other is exnihilated.

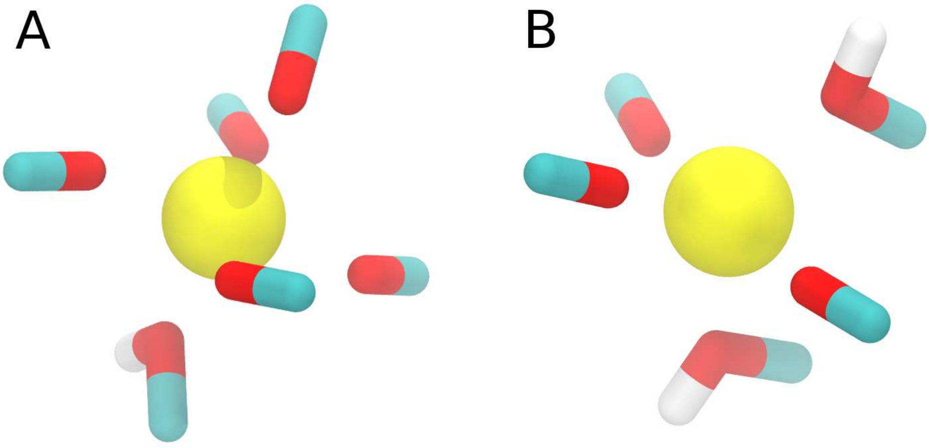

Figure S3: The simplified models representing (A) Na1 and (B) Na2 of LeuT. The positions of the atoms have been optimised from the crystallographic positions, and it is at these points where the additional harmonic constraining potentials are placed. Colours are as for Fig. S2

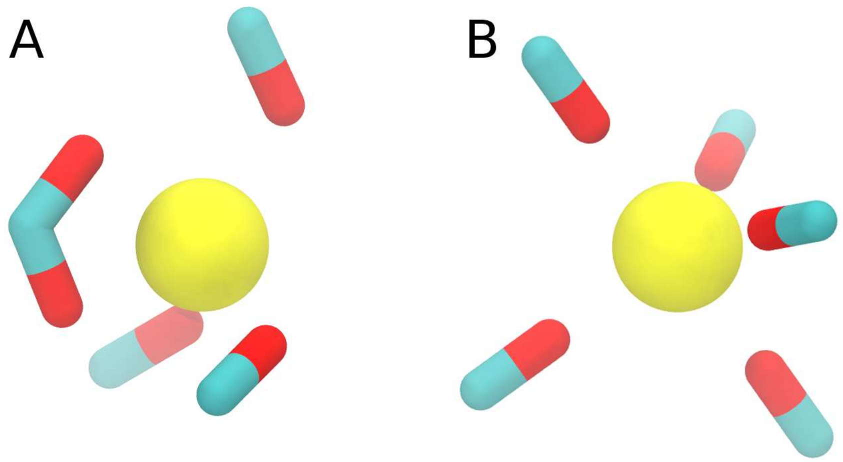

Figure S4: The simplified models representing (A) Na1 and (B) Na2 of Glt_{Ph}. The positions of the atoms have been optimised from the crystallographic positions, and it is at these points where the additional harmonic constraining potentials are placed. Colours are as for Fig. S2

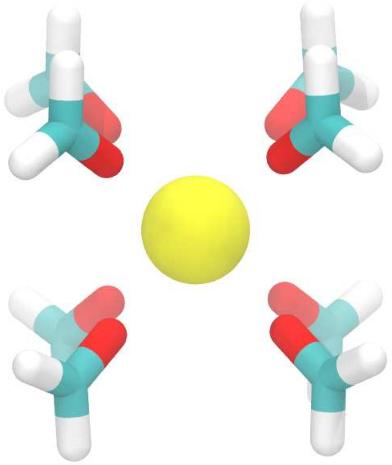

Figure S5: The model representing a K^+ binding site in a potassium ion channel selectivity filter. This model is equivalent to the 8 ligand abstract model. Colours are as for Fig. S2

k (kcal/mol/Å^2)	**Li^+**		**Na^+**		**K^+**	
	$\Delta G_{k\to k=0}$	$\Delta G_{k=0\to k}$	$\Delta G_{k\to k=0}$	$\Delta G_{k=0\to k}$	$\Delta G_{k\to k=0}$	$\Delta G_{k=0\to k}$
**0.001**	0.05	0.24	-0.08	-0.06	0.06	0.30
**0.003**	0.05	0.18	0.06	-0.07	-0.08	0.38
**0.01**	0.44	0.03	0.19	-0.09	0.14	0.14
**0.03**	1.72	-1.54	1.72	-1.90	1.74	-1.47
**0.1**	4.50	-4.49	4.19	-4.23	4.12	-4.00
**0.3**	8.14	-8.27	7.12	-6.94	6.51	-6.40
**1.0**	12.62	-12.56	10.88	-10.93	9.68	-9.54
**3.0**	16.11	-16.04	13.94	-13.90	12.40	-12.27
**10**	18.74	-18.53	16.16	-16.04	14.28	-14.15
**30**	20.02	-20.02	17.17	-17.09	15.09	-14.99
**100**	20.72	-20.88	17.65	-17.78	15.44	-15.35
**300**	20.99	-20.93	17.84	-17.71	15.60	-15.49
**1000**	21.12	-21.02	17.95	-17.87	15.69	-15.60

Table S3: Forward and reverse free energy values (ΔG_1 and ΔG_2) for the 4 ligand RLF abstract model. ΔG values are expressed in units of kcal/mol.

k (kcal/mol/Å^2)	**Li$^+$**		**Na$^+$**		**K$^+$**	
	$\Delta G_{k \to k=0}$	$\Delta G_{k=0 \to k}$	$\Delta G_{k \to k=0}$	$\Delta G_{k=0 \to k}$	$\Delta G_{k \to k=0}$	$\Delta G_{k=0 \to k}$
**0.001**	0.20	0.33	-0.40	0.36	-0.23	0.02
**0.003**	-0.57	0.31	-0.10	0.34	-0.29	0.00
**0.01**	0.18	0.13	0.11	0.25	-0.13	0.19
**0.03**	1.80	-1.39	1.91	-1.50	1.95	-1.60
**0.1**	5.34	-5.06	5.23	-5.12	5.21	-4.89
**0.3**	9.76	-9.55	8.96	-8.86	8.38	-8.21
**1.0**	15.42	-15.20	13.89	-13.77	12.26	-12.17
**3.0**	19.61	-19.50	17.63	-17.53	15.52	-15.45
**10**	22.80	-22.64	20.24	-20.11	17.77	-17.65
**30**	24.42	-24.09	21.46	-21.29	18.66	-18.58
**100**	25.15	-24.99	21.96	-21.82	19.07	-18.80
**300**	25.49	-25.55	22.21	-22.25	19.23	-19.20
**1000**	25.69	-25.73	22.30	-22.38	19.33	-19.06

Table S4: Forward and reverse free energy values (ΔG_1 and ΔG_2) for the 5 ligand RLF abstract model. ΔG values are expressed in units of kcal/mol.

k (kcal/mol/Å^2)	**Li$^+$**		**Na$^+$**		**K$^+$**	
	$\Delta G_{k \to k=0}$	$\Delta G_{k=0 \to k}$	$\Delta G_{k \to k=0}$	$\Delta G_{k=0 \to k}$	$\Delta G_{k \to k=0}$	$\Delta G_{k=0 \to k}$
**0.001**	0.06	0.27	0.13	0.31	0.19	0.30
**0.003**	0.10	0.33	0.10	0.34	0.08	0.46
**0.01**	-0.01	-0.01	0.20	0.22	-0.01	0.31
**0.03**	2.45	-1.86	2.57	-2.66	2.84	-2.41
**0.1**	7.20	-6.83	7.12	-7.02	7.01	-6.60
**0.3**	12.83	-12.69	12.03	-12.01	10.92	-10.52
**1.0**	19.61	-19.42	17.82	-17.73	15.38	-15.02
**3.0**	24.40	-24.22	22.11	-21.99	19.09	-19.01
**10**	27.88	-27.77	25.08	-24.91	21.52	-21.42
**30**	29.73	-29.61	26.33	-26.01	22.51	-22.42
**100**	30.69	-30.47	26.89	-26.58	22.93	-22.84
**300**	31.06	-30.76	27.12	-27.00	23.10	-23.00
**1000**	31.35	-30.95	27.23	-27.11	23.19	-23.10

Table S5: Forward and reverse free energy values (ΔG_1 and ΔG_2) for the 6 ligand RLF abstract model. ΔG values are expressed in units of kcal/mol.

k (kcal/mol/Å^2)	**Li^+**		**Na^+**		**K^+**	
	$\Delta G_{k \to k=0}$	$\Delta G_{k=0 \to k}$	$\Delta G_{k \to k=0}$	$\Delta G_{k=0 \to k}$	$\Delta G_{k \to k=0}$	$\Delta G_{k=0 \to k}$
**0.001**	0.22	0.26	0.30	0.42	0.08	0.42
**0.003**	-0.06	0.31	-0.15	0.52	-0.21	0.65
**0.01**	-0.52	0.37	-0.13	0.50	0.11	-0.24
**0.03**	1.79	-1.11	2.08	-1.61	2.43	-2.86
**0.1**	5.44	-5.18	6.84	-6.53	7.15	-6.73
**0.3**	9.54	-9.38	11.94	-11.70	11.67	-11.30
**1.0**	13.70	-13.44	18.16	-17.77	16.77	-16.52
**3.0**	15.26	-15.05	23.03	-22.67	20.78	-20.59
**10**	13.62	-13.40	26.53	-26.13	23.58	-23.33
**30**	9.98	-9.61	28.18	-27.79	24.73	-24.48
**100**	5.71	-5.37	28.98	-28.59	25.25	-25.03
**300**	2.83	-2.47	29.27	-29.08	25.43	-25.21
**1000**	0.73	-0.40	29.42	-29.22	25.52	-25.30

Table S6: Forward and reverse free energy values (ΔG_1 and ΔG_2) for the 7 ligand RLF abstract model. ΔG values are expressed in units of kcal/mol.

k (kcal/mol/Å^2)	**Li$^+$**		**Na$^+$**		**K$^+$**	
	$\Delta G_{k\to k=0}$	$\Delta G_{k=0\to k}$	$\Delta G_{k\to k=0}$	$\Delta G_{k=0\to k}$	$\Delta G_{k\to k=0}$	$\Delta G_{k=0\to k}$
**0.001**	0.15	0.50	-0.15	0.64	-0.20	0.40
**0.003**	0.19	0.75	0.12	0.83	0.14	0.71
**0.01**	0.14	1.00	-0.06	1.00	0.07	0.86
**0.03**	1.54	-1.10	2.24	-1.60	0.14	0.29
**0.1**	5.57	-4.89	7.12	-7.10	8.44	-7.79
**0.3**	9.03	-8.58	12.27	-11.72	13.55	-13.42
**1.0**	9.33	-8.86	17.92	-17.43	18.42	-17.68
**3.0**	2.91	-2.45	20.50	-19.90	21.90	-21.19
**10**	-14.75	15.19	21.74	-21.46	24.38	-23.62
**30**	-38.24	38.76	22.52	-21.86	25.45	-24.76
**100**	-62.79	63.29	22.97	-22.57	25.93	-25.80
**300**	-81.92	82.09	23.15	-22.81	26.09	-25.41
**1000**	-98.49	98.73	23.17	-22.73	26.21	-26.05

Table S7: Forward and reverse free energy values (ΔG_1 and ΔG_2) for the 8 ligand RLF abstract model. ΔG values are expressed in units of kcal/mol.

k (kcal/mol/Å^2)	**Li^+**		**Na^+**		**K^+**	
	$\Delta G_{k \to k=0}$	$\Delta G_{k=0 \to k}$	$\Delta G_{k \to k=0}$	$\Delta G_{k=0 \to k}$	$\Delta G_{k \to k=0}$	$\Delta G_{k=0 \to k}$
**0.001**	0.41	0.37	-0.24	0.07	0.15	0.32
**0.003**	0.43	0.16	0.23	-0.17	-0.25	0.20
**0.01**	0.27	0.16	0.15	-0.28	0.08	0.50
**0.03**	0.23	0.19	0.69	-0.54	0.47	0.21
**0.1**	2.90	-2.18	3.25	-2.97	2.75	-2.42
**0.3**	6.87	-6.48	7.50	-7.11	6.04	-6.04
**1.0**	13.15	-12.77	13.21	-13.05	10.96	-10.69
**3.0**	19.07	-18.87	18.73	-18.47	15.80	-15.67
**10**	24.06	-23.92	23.20	-23.25	19.71	-19.40
**30**	26.93	-26.59	25.56	-25.67	21.77	-21.70
**100**	28.53	-28.36	26.74	-26.5	22.80	-22.5
**300**	29.19	-29.69	27.22	-27.02	23.18	-22.91
**1000**	29.45	-29.41	27.37	-27.32	23.29	-23.06

Table S8: Forward and reverse free energy values (ΔG_1 and ΔG_2) for the RLF LeuT:Na1 model. ΔG values are expressed in units of kcal/mol.

k **(kcal/mol/Å^2)**	**Li$^+$**		**Na$^+$**		**K$^+$**	
	$\Delta G_{k \to k=0}$	$\Delta G_{k=0 \to k}$	$\Delta G_{k \to k=0}$	$\Delta G_{k=0 \to k}$	$\Delta G_{k \to k=0}$	$\Delta G_{k=0 \to k}$
**0.001**	0.07	0.07	-0.10	0.10	0.24	0.23
**0.003**	0.09	0.08	0.04	0.19	0.08	-0.51
**0.01**	0.12	0.14	0.12	0.00	-0.36	-0.47
**0.03**	0.60	-0.35	0.75	-0.26	0.47	-0.05
**0.1**	2.58	-2.33	2.95	-2.64	2.80	-3.24
**0.3**	6.49	-6.51	6.13	-6.03	5.65	-5.38
**1.0**	12.48	-12.20	11.44	-11.4	9.85	-9.55
**3.0**	18.01	-18.25	16.32	-16.34	13.96	-13.69
**10**	22.45	-22.51	20.16	-20.16	17.33	-17.13
**30**	24.91	-24.70	22.12	-22.12	19.02	-18.73
**100**	26.30	-26.06	23.13	-22.96	19.79	-19.62
**300**	26.84	-26.68	23.51	-23.28	20.1	-19.91
**1000**	27.04	-27.45	23.69	-23.48	20.24	-20.06

Table S9: Forward and reverse free energy values (ΔG_1 and ΔG_2) for the RLF LeuT:Na2 model. ΔG values are expressed in units of kcal/mol.

k (kcal/mol/Å^2)	**Li$^+$**		**Na$^+$**		**K$^+$**	
	$\Delta G_{k\to k=0}$	$\Delta G_{k=0\to k}$	$\Delta G_{k\to k=0}$	$\Delta G_{k=0\to k}$	$\Delta G_{k\to k=0}$	$\Delta G_{k=0\to k}$
**0.001**	0.04	0.10	0.10	0.11	0.14	-0.20
**0.003**	-0.17	0.23	-0.01	0.00	0.07	-0.15
**0.01**	0.30	0.08	0.08	0.09	0.13	0.07
**0.03**	0.85	-0.43	0.50	-0.18	-0.30	0.14
**0.1**	2.30	-2.05	2.03	-1.99	1.79	-1.45
**0.3**	5.13	-5.04	4.57	-4.42	4.32	-4.03
**1.0**	9.55	-9.42	8.60	-8.81	8.18	-7.61
**3.0**	14.25	-14.23	12.87	-12.84	12.13	-11.49
**10**	18.22	-18.00	16.57	-16.54	15.68	-14.74
**30**	20.68	-20.56	18.91	-18.68	17.83	-16.84
**100**	22.54	-22.88	20.41	-20.22	19.11	-17.98
**300**	23.92	-23.78	21.23	-21.06	19.70	-18.60
**1000**	24.76	-24.77	21.78	-21.76	20.13	-19.03

Table S10: Forward and reverse free energy values (ΔG_1 and ΔG_2) for the RLF Glt_{Ph}:Na1 model. ΔG values are expressed in units of kcal/mol.

k (kcal/mol/Å^2)	**Li$^+$**		**Na$^+$**		**K$^+$**	
	$\Delta G_{k \to k=0}$	$\Delta G_{k=0 \to k}$	$\Delta G_{k \to k=0}$	$\Delta G_{k=0 \to k}$	$\Delta G_{k \to k=0}$	$\Delta G_{k=0 \to k}$
**0.001**	0.04	0.05	0.01	0.26	0.21	-0.02
**0.003**	0.22	0.22	0.14	0.22	-0.10	0.07
**0.01**	0.40	-0.09	0.22	0.22	-0.10	0.43
**0.03**	0.63	-0.23	0.50	-0.18	0.33	-0.11
**0.1**	2.52	-2.29	2.43	-2.22	2.56	-2.18
**0.3**	6.22	-5.78	5.71	-5.58	5.38	-5.16
**1.0**	11.69	-11.48	10.53	-10.39	9.28	-9.10
**3.0**	16.85	-16.97	15.24	-15.24	13.31	-13.12
**10**	21.34	-21.12	19.03	-18.71	16.69	-16.43
**30**	23.83	-23.59	21.05	-20.93	18.37	-18.23
**100**	25.13	-25.14	22.07	-21.85	19.20	-19.10
**300**	25.63	-25.55	22.38	-22.39	19.48	-19.29
**1000**	25.97	-25.87	22.55	-22.57	19.59	-19.46

Table S11: Forward and reverse free energy values (ΔG_1 and ΔG_2) for RLF Glt_{Ph}:Na2 model. ΔG values are expressed in units of kcal/mol.

**Model**	**Site**	**$Na^+ \rightarrow Li^+$**	**$Li^+ \rightarrow Na^+$**	**$Na^+ \rightarrow K^+$**	**$K^+ \rightarrow Na^+$**
**Abstract**	**4 Ligands**	-22.03	22.29	14.60	-14.62
	**5 Ligands**	-20.55	20.48	15.63	-15.78
	**6 Ligands**	-19.27	19.10	15.87	-16.06
	**7 Ligands**	-16.89	17.21	14.35	-14.35
	**8 Ligands**	-16.18	16.18	12.73	-12.60
**LeuT**	**Na1**	-22.70	22.59	18.30	-18.33
	**Na2**	-21.47	21.53	17.03	-17.10
**Glt_{Ph}**	**Na1**	-29.35	29.33	22.48	-22.47
	**Na2**	-20.58	20.59	15.92	-15.92

Table S12: Forward and reverse ΔG_{Ex1} values (see equation 3). ΔG values are expressed in units of kcal/mol.

References

[1] Yamashita A, Singh S, Kawate T, Jin Y, Gouaux E (2005) Crystal structure of a bacterial homologue of Na^+/Cl^--dependent neurotransmitter transporters. Nature 437: 215-223.

[2] Noskov S, Roux B (2008) Control of ion selectivity in LeuT: two Na^+ binding sites with two different mechanisms. J Mol Biol 377: 804-818.

[3] Boudker O, Ryan R, Yernool D, Shimamoto K, Gouaux E (2007) Coupling substrate and ion binding to extracellular gate of a sodium-dependent aspartate transporter. Nature 445: 387-393.

[4] Thomas M, Jayatilaka D, Corry B (2011) Mapping the importance of four factors in creating monovalent ion selectivity in biological molecules. Biophys J 100: 60-69.
